# A Design Framework of Medical Wayfinding Signs for the Elderly: Based on the Situational Cognitive Commonness

**DOI:** 10.3390/ijerph192113885

**Published:** 2022-10-25

**Authors:** Jianfeng Wu, Xinyu Liu, Chunfu Lu, Shihan Yu, Dongfang Jiao, Xinyu Ye, Yuqing Zhu

**Affiliations:** 1 Industrial Design and Research Institute, Zhejiang University of Technology, Hangzhou 310023, China; 2 School of Design and Architecture, Zhejiang University of Technology, Hangzhou 310023, China

**Keywords:** cognitive commonness, situation theory, wayfinding sign, elderly, geriatric hospital, medical signs, design framework

## Abstract

Older people in China have a poor understanding of hospital signage. To address this problem, in this study, we combined the theories of situated cognition and cognitive commonness in order to introduce the three main factors that affect the generation of situational cognitive commonness: composition of the situation, familiarity, and concreteness. We used these theories to construct a methodological framework for the design of geriatric hospital wayfinding signs that were based on situational cognitive commonness. The design of nine healthcare signs for Chinese national standards were used as examples in the study. First, users who were familiar with medical scenarios were asked to draw concrete cognitive conception graphics for the purposes of individual wayfinding targets from both physical and social situations. Next, we coded and grouped the generated graphics based on their situational features in order to extract groups of representative common graphics. Finally, we reorganized the common graphics and developed concrete designs, which were tested by the judgment test. The wayfinding signs designed according to the methodological framework of this study effectively improved the understanding of hospital signage among older Chinese people. This study took geriatric hospital wayfinding signs as the examples to provide a feasible theoretical basis and research reference for symbol design.

## 1. Introduction

Healthcare signage uses graphics as the primary feature to provide guidance [1]. Older patients use these signs to help them with wayfinding when seeking medical treatments [2,3]. In order to enhance the understanding of healthcare signage among older patients, many researchers have constructed in-depth studies on the effective expression of visual features in healthcare signs [4,5,6] and factors that affect the understanding of graphics among older people [6,7,8]. Due to differences in understanding and the cognition of symbols between designers and elderly users [2], the existing healthcare and hospital signage is still not friendly to the elderly.

To minimize the cognitive gap between designers and specific user groups, some studies have proposed symbol design methods based on the principle of user participation [2,9,10]. According to this principle, users can draw on their ideas about the symbols and designers analyze these ideas to extract representing graphics, which have “cognitive commonness”—that is, constrained by the experience of the objective world, the cognitions of a specific group of people share certain characteristics and patterns, which reflect the common cognitive structure of the group [11,12,13]. Finally, these graphics can be optimized to generate symbols that will best enhance user understanding [14,15,16]. Although it has been shown that the graphical expression may be affected by multiple factors, such as the users’ ability to present their ideas in graphics [17], education level [18], experience [7], and age [19], users influenced by these factors can still provide valuable ideas for symbol design [17]. These methods have been used extensively for public symbol designs, such as transportation [20], tourism [21], and healthcare [22]. When these methods are used, the degree that cognitive commonness is generated is generally determined by the ratio of the number of common graphics to the total number of graphics drawn by users. The graphic category with the highest number of common ideas represents the users’ cognitive commonness, which is also the chief source of the graphics used for symbol design [23]. However, it is not enough to take “the number of similar graphics” as the standard to obtain users’ cognitive commonness, due to the fact that there is no way to ensure that these graphics are common and representative for a specific population. In addition, the lack of constraints on the semantics of the graphics when extracting common ideas may limit the comprehensibility of the redesigned symbols [7]. Thus, further research is needed to summarize the common ideas in order to extract users’ cognitive commonness.

Some researchers have studied the comprehension of symbols from the perspective of situated cognition [24,25]. The findings indicated that users have a better understanding of symbols that represent a complete application situation [26], suggesting a new perspective for the interpretation of accurately extracting cognitive commonness. In this study, we introduced the theory of situated cognition in order to construct a methodological framework for symbol design based on the situational cognitive commonness. We also selected signs used for Chinese geriatric hospital wayfinding in order to carry out the design and to evaluate and discuss the methods.

## 2. Related Research

### 2.1. Symbol Design Based on Cognitive Commonness

Previous studies have shown that cognitive convergence exists between groups. This convergence is not only reflected in the form of information storage and processing, but also has an important impact on the output of cognitive activities. Some researchers have used the concept of cognitive commonness to describe this phenomenon [11,12,13]. Further, this theory of cognitive commonness has raised significant attention in the field of graphic design [10,15,27]. First, it has been used to visualize cognitive expression by graphics in order to eliminate the cognitive friction that exists between designers and users and also to reduce the perceived challenge in design tasks and design expression. Second, the expected preferences for specific symbols are inferred by illustrating the common ideas of the users, providing references for the design of the symbols. For example, Ng [17] explored the cognitive commonness of older people and collected their ideas on the signage used in public. They then summarized and evaluated the common ideas of the older people. Green [15] guided users to draw graphics that symbolize the functions of automotive instrument panels, and then extracted the common graphics to create a candidate set of automotive symbols. The question of how to accurately obtain and extract the cognitive commonness of users, however, remains a core challenge when applying these methods. Existing research has mainly adopted the measurement of an absolute number of common ideas and considered the category of ideas that accounts for the largest proportion of ideas of a user group as cognitive commonness [23,28]. Although using an absolute number of common ideas to determine cognitive commonness is simple and easy to implement, this approach can also be affected by many factors, such as the users’ drawing ability, object imagery preference [17], gender, and age [7,19]. These user factors may result in deviations in these ideas. Therefore, further research is required to ensure the accuracy and perfection of the graphics used to describe and represent cognition and ideas.

### 2.2. The Theory of Situated Cognition and Cognitive Commonness

According to the theory of situated cognition, cognition occurs in complex social contexts involving people, actions, and situations [29,30]. Rambusch [31] and Echterhoff [32] showed that information that presents situational features is more likely to be transmitted among groups and tends to develop a cognitive commonness among groups. The more specific the situational features, the better the ability of individuals to imagine and remember the relevant information, which leads to higher cognitive similarity of the shared information [33]. For example, Lesch [24] showed that scenario training not only improved the cognitive levels and understanding of warning symbols among older people, but also strengthened the ability of older people to correctly respond to the hazards indicated by the warning symbols. The results of Lesch’s study provided a new idea for drawing graphics representing cognitive commonness—that is, the use of situational elements as the basis for drawing and extracting common ideas based on cognitive commonness.

The findings of the above studies suggest that three main factors affect the situational cognitive commonness:

1. Composition of the situation. The human cognitive system is suprapersonal; cognition is constructed and stored in the brain and affected by physical activities and the environment of social interaction [31,34]. Hutchins [35] showed that completion of a flight required information processing in the brain of pilots, their usage of physical tools in the cockpit to store the information, and thus researchers explain pilots’ behavioral attributes through their situational representation of tool use. Rizzolatti [36] proposed that human cognition and behaviors are affected by a matching system that exists between action execution and action observation in social situations. In a specific situation, human cognition originates from perceptual activities, depending on the contact with physical equipment and physical structures in the environment as well as in the social interactions (behaviors, language, and other communication methods) held with other people. Both are important components in situated cognition.
2. Familiarity. Cognitive commonness is affected by familiarity [37], which promotes the generation of more information related to situated cognition. Increasing evidence has supported the idea that individuals who are more familiar with the items and events they need to describe are capable of constructing a richer situational model [38]. The theory of situated cognition suggests that the more familiar an individual is with the target information, the more likely it is that he/she can process the situational representations based on memory [30]. A study by Shen [37] showed that people who are familiar with a restaurant, for example, could retrieve the situational information of the restaurant’s service from their memory, which directly affected their perception of the restaurant’s service. Another study by Shen [39] showed that a user’s familiarity with the objects depicted in a symbol affected their understanding of that symbol, suggesting that designers should use patterns and elements that are familiar to the users. Green [15] believed that it was difficult for individuals to draw symbols to represent unfamiliar situations during graphic generation. Selecting individuals who were more familiar with the target information to draw the symbols improved the efficiency of graphic design. In other words, the more familiar the users are with a specific interaction and scenario, the easier it is to recall their relatively complete memory of that situation. These users can build a rich situational-cognitive model by recalling the situational features in the brain, so that they can produce more situational information that is common. As a result, designers can extract the ideas of situational cognitive commonness among the users.
3. Concreteness. When the ideas that symbols represent are more specific and simpler, the semantics are clearer and easier to understand [40]. It is easier for people to generate concrete graphics of cognitive commonness [17] and to actively use concrete elements to create patterns. For example, Picard [41] believed that children’s drawings conveyed information about objects or scenes in concrete forms. Jones [42] showed that older people tended to show concrete elements with less complexity in cognition and visual processing in their drawings and that they are good at drawing concrete graphics. Hence, selection of specific and authentic situational information that represents a familiar environment is needed when designing symbols. In addition, the visual elements should be simplified but should not be too abstractive in order to allow users to have a consistent understanding.

## 3. Methodological Framework for Symbol Design and Operating Procedures Based on the Situational Cognitive Commonness

### 3.1. Framework for Symbol Design

**Figure 1:**
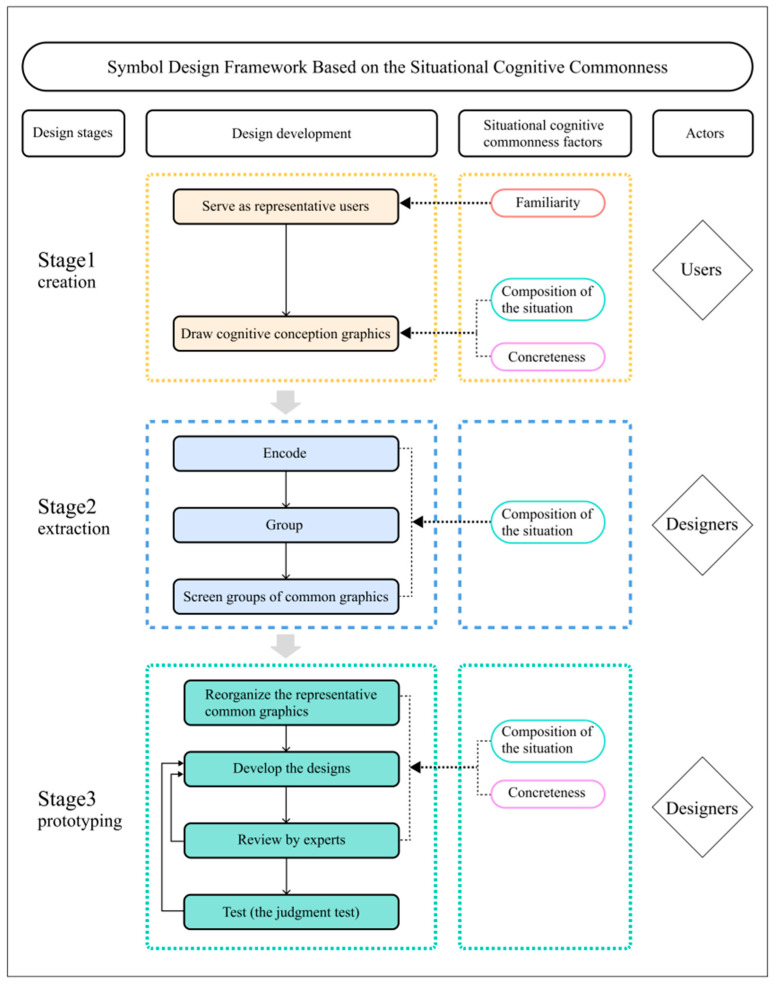
Symbol design framework based on the situational cognitive commonness.

As mentioned above, situated cognition reflects the cognitive commonness of users. As such, this study proposed a symbol design framework that is based on situational cognitive commonness (Figure 1). This framework was based mainly on a previous report by Green [15]. We embedded the theory of situated cognition into the symbol design framework, which is based on situational cognitive commonness. At both the user and designer levels, we introduced the three factors (i.e., composition of the situation, familiarity, and concreteness) that affect situational cognitive commonness into all three graphic design stages (i.e., creation, extraction, and prototyping). The study focused on how to establish cognitive commonness that is based on situational measures for the purposes of the better design of symbols.

At the user’s level, all three factors (i.e., composition of the situation, familiarity, and concreteness) were fully utilized. Familiarity with the situation enabled users to build a rich situational-cognitive model, thereby improving the creation of the ideas for situational cognitive commonness. The brain recalled the situational features experienced by the users and presented them in concrete forms. During the first stage (i.e., creation) of symbol design, researchers screened representative users who were familiar with the wayfinding target situations. For example, people who visited a place several times in a certain period of time were familiar with the situation of this space, and therefore had advantages in their situated cognition when conceiving graphics. As such, the designers promoted them to draw graphics from both social and physical situations and requested the users to use concrete expressions in order to create cognitive conception graphics.

At the designer’s level, the two factors of composition of the situation and concreteness were introduced. The second (extraction) stage involved the composition of the situation. Situational elements, as the bases for extracting common ideas and the references for the prototyping stage, affected the effectiveness of the proposed designs. Designers encoded the graphics generated by the users and grouped the graphics based on their physical and social situational elements. The designers subsequently screened groups of common graphics based on the graphics of the groups and the number of common ideas. Both composition of the situation and concreteness were introduced during the prototyping stage of symbol design. Situational information triggers the cognitive commonness of specific events and items in humans. The concrete expression of ideas helped users to easily understand the graphics. Therefore, concrete expression was regarded as a tool to assist situational design. At the prototyping stage, designers may prioritize the elements of common graphic groups, and screen the graphics with higher importance for design. The designers supplemented the physical and social situational elements that had been missing in the groups of common graphics, which was followed by the development of the symbols in a concrete form according to the design criteria.

### 3.2. Operating Procedures

During the first stage (i.e., creation), cognitive conception graphics of optimized symbols were created by the users. First, for symbols that were not easily understood by the target users, experimental materials (e.g., title, meaning, and drawing region) were generated in the designated area. Second, users who were familiar with the application situation of the symbols were invited to participate in graphing and were guided to imagine the personnel, objects, environments, and human interaction scenarios. It should be noted that the researchers instructed the participants to recall the wayfinding target situations rather than the wayfinding symbols in order to simulate situational cognition. The symbol names were given to the users as textual stimuli, and the users were required to draw concrete graphics. The users were interviewed about their drawing ideas during or after they had completed drawing.

During the second stage (i.e., extraction), the graphics created by the users were extracted in order to form graphics of cognitive commonness for subsequent design use. First, the designers needed to eliminate the drawing papers that possessed no responses or only plain text responses. Further, the designers then carefully followed the principle of extracting cognitive commonness with situational features by encoding the user-drawn graphics that were based on situational features, such as interaction scenarios and related devices. Subsequently, they divided the graphics into groups, then screened and selected the groups that met the situational features, and that also had a relatively large number of responses, in order to form groups of the common graphics. As the participants’ graphics may not be intuitive enough, designers in this stage were required to be aware of the wayfinding target location beforehand in order to increase their familiarity with the surroundings. They were required to know exactly what each idea was meant to represent and to interact with the participants more.

During the third stage (i.e., prototyping), the symbols were designed and tested according to the groups of common graphics. The designers were required to follow the requirements of concreteness and situationalization in order to extract and reorganize the representative graphics from the groups of common graphics. The key to this link was such that the scene represented by the graphics should be easy to understand in order to reveal the intent of the application of the symbols in the actual scenario. Various graphics were integrated in order to express the key elements and interactions in the scenario. Designers prioritized the graphic elements of each group according to their importance, and then extracted the elements that met the method standards and were easy to express as representative graphics. Designers used had rich experience in wayfinding target situations, so that they could supplement the situational elements that the users might have missed, and to also aid the users through drawing design sketches. Standard symbols, design specifications, and users’ visual habits and cognitive rules were subsequently combined to form standardized symbol designs. Then, the designs were reviewed and revised by experts in order to form symbols that were based on the users’ situational cognitive commonness. Finally, participants who were not involved in the design were invited to complete the judgement test developed by the International Organization for Standardization [43] in order to examine differences in the user’s understanding of the symbols before and after the design. The two symbol variants with the same meaning from before and after the optimization were placed in one group in a random order and labeled. The symbols in each group were arranged from top to bottom and were printed on a piece of white paper as experimental materials. According to the judgment test criteria, the comprehension of each symbol variant was determined by estimating the percentage of the total population who understood the correct meaning of the symbol variant [2,44]. All participants were required to write a judgment value in the blank space next to each symbol variant.

## 4. Application of Symbol Design Method Based on the Situational Cognitive Commonness

To test the effectiveness of the symbol design framework (which was based on the users’ situational cognitive commonness), we carried out a design practice for geriatric hospital wayfinding signs in China. Although healthcare signs are widely used, they are not universally understood by older people. Hence, redesigning these healthcare symbols may help improve the problem of symbol cognition among older people. This design method was also evaluated and improved in this practice. The steps associated with the symbol design approach, based on situational cognitive commonness, are discussed in the following sections.

### 4.1. Design Preparation

**Figure 2:**
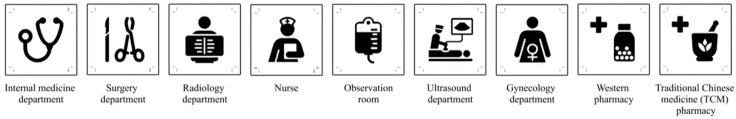
Healthcare wayfinding signs developed by the National Standard of the People’s Republic of China GB/T 10001.6-2021.

**Figure 3:**
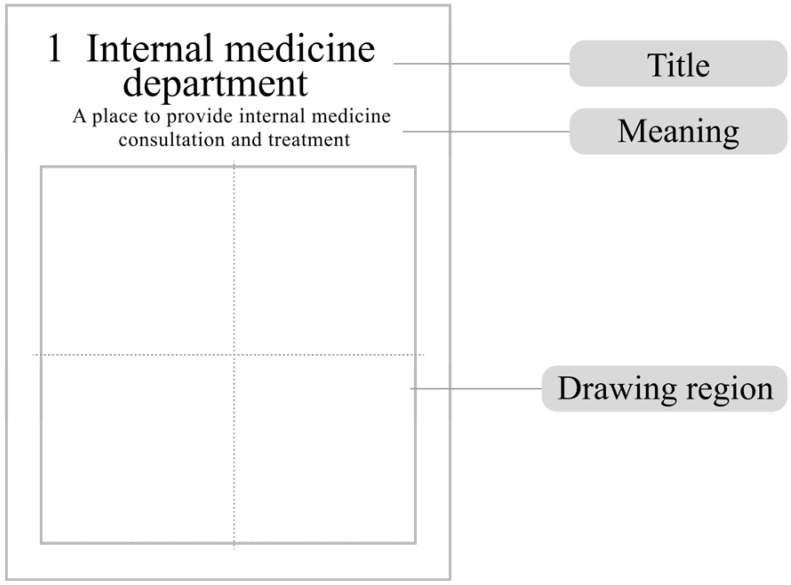
Presentation of a representative experimental material. Note: The experimental material for users’ drawing of symbol for the “internal medicine department” was taken as an example.

The symbols for medical treatment and healthcare regulated by the National Standard of the People’s Republic of China [45] were implemented only in October 2021. As there was no report on public identification and testing, we selected nine wayfinding signs for healthcare as experimental materials in this study. These signs showed basic information about some of the major departments in a healthcare environment, including “internal medicine department”, “surgery department”, “radiology department”, “nurse”, “observation room”, “ultrasound department”, “gynecology department”, “western pharmacy”, and “traditional Chinese medicine (TCM) pharmacy”. Figure 2 shows the specific symbols and names selected in this study. Figure 3 shows an example of the experimental materials, including a drawing paper for users at the first (creation) stage that contains a title, a description of the title, and a drawing area of 8 × 8 cm^2^. The drawing area is divided into 2 × 2 squares by the central axes.

### 4.2. Obtaining Ideas from the Users

This study recruited participants from several communities in Hangzhou City, Zhejiang Province, China, and all of the research protocol for this study was approved by the Ethics Committee of the Institute of Industrial Design of Zhejiang University of Technology (Zhejiang Province, China). All participants gave their verbal informed consent before participating the experiment.

In order to obtain, relatively, enough common ideas, we used the information saturation sampling principle for reference [46]. As such, a sequential participant experiment format was adopted. The first stage of graphic design (i.e., creation) enrolled a total of 24 older people, which included 14 men and 10 women, aged 73 ± 9.7 years on average (mean ± standard deviation). All participants had elementary school education or above, and their backgrounds were in cities. These 24 participants were told that this part of the experiment would last for 20–30 min, and that they would receive a material reward at the end of the experiments. These participants had received medical treatments frequently and had also visited hospitals more than five times in the past year. They also had a high degree of familiarity with healthcare environments in general and were representative of the population.

During the experiments, these participants were provided paper materials and a pencil. They were then instructed to read each title (e.g., medical department, service, etc.) and to imagine each situation. They were then asked to draw a picture of the situation described and to complete the drawing independently. Participants were allowed to write or draw in the drawing area, without a right or wrong answer. Participants were encouraged to verbally describe their drawing ideas during the drawing task. Their thoughts were recorded by the researchers. All participants were interviewed by the researchers after they had completed their drawing. Participants who were not able to complete the drawing task were allowed to withdraw from the study, and their experimental data were not used for subsequent analysis.

### 4.3. Extracting the Common Graphics

**Figure 4:**
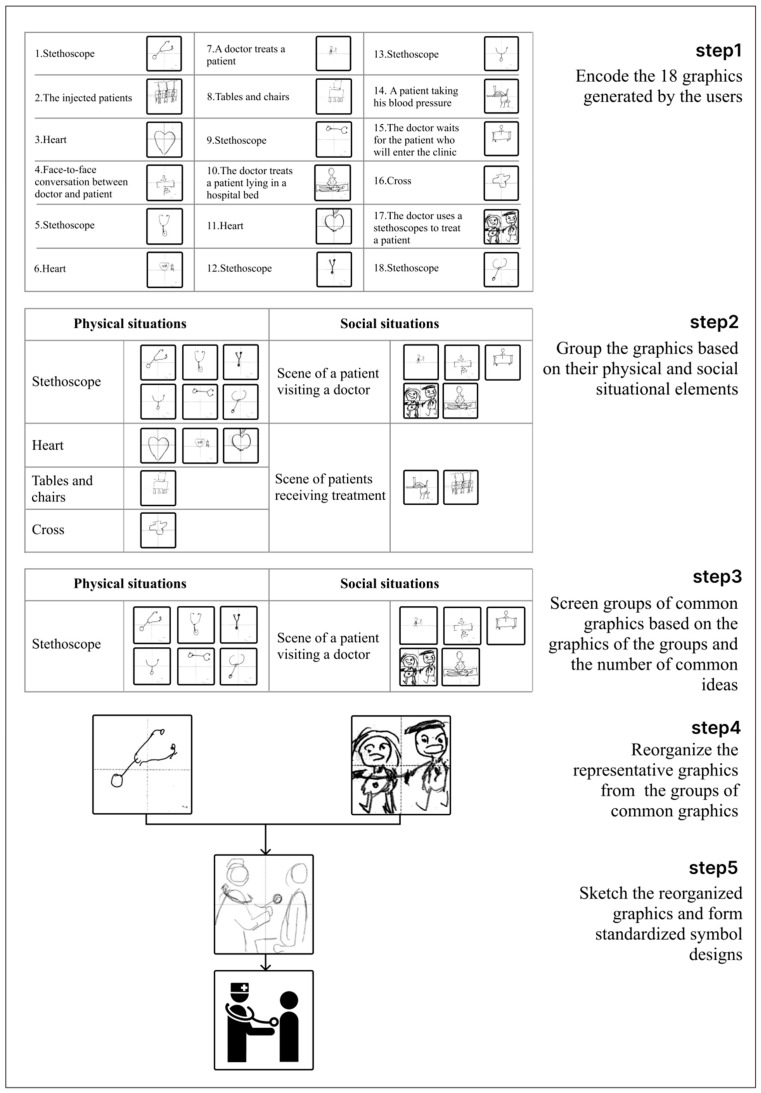
Extraction and prototyping of healthcare symbols for the “internal medicine department” and the development of a design.

After eliminating the drawing papers with no responses or only plain text responses, a total of 174 graphics for the nine symbols were obtained at the end of the first stage (i.e., creation). Both pure graphics and graphics marked with a small amount of text were defined as valid responses. Figure 4 shows the process of extracting the common graphics for a healthcare symbol, specifically for a “internal medicine department”, and the developing of the designs. The designers had to accurately extract the common graphics. They combined the recorded ideas of the individual users with their drawings in order to encode the graphics that were based on physical situations (e.g., characters involved in the situations, medical equipment, and medical environments) as well as social situations (e.g., doctor–patient interaction scenarios) and subsequently divided the drawings into six groups. Among these six groups of drawings, the “stethoscope” group and “scene of a patient visiting a doctor” group contained obvious situational features and had many graphic responses. Therefore, these two groups were extracted from the six groups to form a group of common ideas under the title of the “internal medicine department”.

### 4.4. Output of Symbol Design

In this study, the output was a symbol design that represented the cognitive commonness of the users. The designers restored the scenario of interaction based on both the physical and social situations, and then explored which situational elements the users may encounter. Subsequently, they extracted the representative graphic elements (e.g., doctor, patient, and stethoscope) from the groups of common graphics (e.g., “scene of a patient visiting a doctor” and “stethoscope”). The designers reorganized the graphics and supplemented the situational elements that the users might have missed, and then sketched the designs. A person wearing a surgical scrub cap and carrying a stethoscope represented a doctor, and a commonly used human pattern was used to represent a general patient. The stethoscope was drawn in the form of circles and arcs and the characteristics of the stethoscope were properly amplified to draw users’ attention and facilitate understanding. In addition, the symbol featured the scenario information of a doctor using the stethoscope to examine a patient who was in a seated position. After finishing the sketch, we used Adobe Illustrator and Adobe XD to develop the actual symbols based on these sketches.

**Figure 5:**
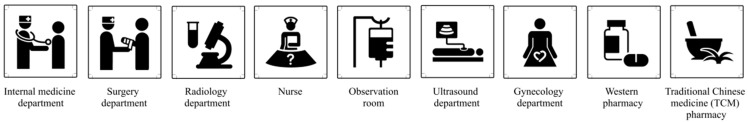
The designs of healthcare wayfinding signs in this study.

After all designs were developed, three experts were invited to comment on the designed symbols. After several rounds of revisions based on the experts’ opinions, the final “internal medicine department” design was obtained. Figure 5 shows the designs of the nine healthcare symbols that were developed in this study.

### 4.5. Design Test and Statistical Analysis

To verify the effectiveness of the symbol design methods based on situational cognitive commonness, we verified the differences in understanding the healthcare wayfinding signs before and after optimization of the designs by the judgment test.

This study recruited 77 Chinese older men (44) and women (33), who were all older than 60 years old, from several communities in Hangzhou City to participate in the design test. All representative participants had an education level of elementary school or above, and all grew up and lived in the city. None of them had severe cognitive or visual impairment, nor participated in the graphic drawing task (experiments listed in Section 4.2). All were non-medical-related professionals who received a monetary reward at the end of the experiments.

**Figure 6:**
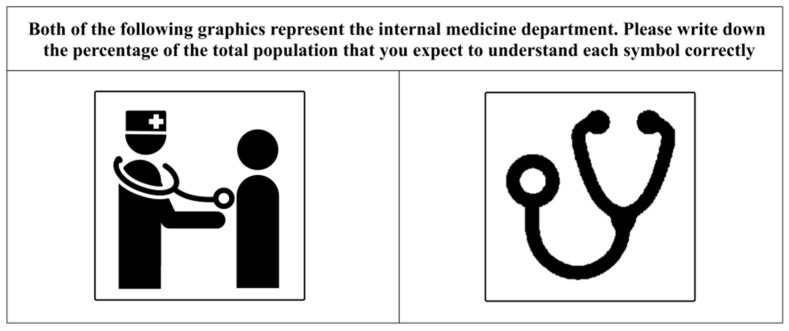
Examples of symbol materials for the judgment test. Note: The materials for the “internal medicine department” here are taken as examples.

The experimental materials included printed paper materials containing nine groups of healthcare symbols before and after optimization (representative examples are shown in Figure 2 and Figure 5), with the symbol titles and descriptions marked on the top of the papers (Figure 6). Two symbol variants (with the same meaning) before and after optimization were put in the same group and were randomly arranged in the left and right columns. According to the judgment test criteria, these 77 participants were required to estimate the percentage of the total population (0–100%) that may understand each symbol variant correctly [2,44], followed by writing the judgment value (estimated percentage of the total population) in the blank space next to each symbol variant.

To test whether the design method based on the situational cognitive commonness could improve symbol understanding among older users, this study used a paired *t*-test to analyze the data and to compare the understanding before and after optimization of the designs. An alpha of 0.05 was used to test significance in this study.

The results of the paired *t*-test showed that the average understanding of six out of nine optimized symbols was significantly higher than that of the existing designs, including the “internal medicine department”, “surgery department”, “radiology department”, “nurse”, “observation room”, and “gynecology department” (Table 1). Among the remaining three schemes, no significant difference was found before and after optimization for the “western pharmacy” and “TCM pharmacy” symbols, whereas the average understanding of the optimized symbol for “ultrasound department” was slightly reduced. Therefore, among the nine design schemes, a total of six symbols provided better understanding to the users, compared with the existing national standard graphic symbols. This result suggested that our symbol design method based on the situational cognitive commonness was effective in improving the understanding of graphic symbols among older Chinese people.

## 5. Discussion

### 5.1. Major Findings

#### 5.1.1. Role of Situated Cognition in Symbol Design

This study integrated the theory of situated cognition in a symbol design method based on situational cognitive commonness. Users and designers were guided to use situational representation in the physical and social situations during three different stages of graphic design: creation of cognitive graphics, extraction of common graphics, and design output. Among the nine new healthcare symbols with situational elements in the assessments, six of them (i.e., “internal medicine department”, “surgery department”, “radiology department”, “nurse”, “observation room”, and “gynecology department”) achieved significantly better user understanding after optimization, indicating the necessity of introducing situated cognition into symbol design. As mentioned earlier, when extracting common ideas from a user group, the category with the highest percentage of cognitive ideas was typically used to determine cognitive commonness [23,28], which may, however, lack constraint on graphic semantics when extracting common ideas [7]. In this study, designers used users’ high cognitive similarity to medical situational features in order to direct users in the generation and evaluation of symbols [33]. The users’ cognition of healthcare symbols was associated with users’ spatial interaction with medical treatment, and their memories of healthcare scenarios were stimulated by situational visual representations. The commonness and uniformity of healthcare scenarios led to cognitive convergence in older people. In accordance with cognitive theories, they created graphs with the same or with similar semantics; with this approach, then, the understanding of the optimized medical symbols can be improved.

This finding was also supported in a study by Patel [22], which used the Snellen eye chart, ophthalmologist graphics, and eye graphics in order to redesign the ophthalmologist icon for hospitals in central India. By fully presenting the characters, equipment, and elements of the ophthalmology clinic, they effectively improved the users’ understanding of the icons.

In addition, our study also showed that older people’s judgments of symbols were not only determined by their specific representations of the situation, but were also influenced by long-term aesthetic preferences and stereotypes. For example, the symbol of “gynecology department” was composed of elements for a woman and a heart (love). Although this symbol did not present the medical situation of a gynecology clinical, it still evoked a high level of understanding by the older user, which was consistent with the finding of Smith [47]. This result also confirmed the report of Schwarz [30] that people relied on general knowledge of items and events and tended to form judgments in a top-down manner. In this study, old Chinese people already had deep-rooted cognitive standards for the symbol of the “gynecology department”. Even if the situational features were not obvious in this circumstance, older Chinese people still made judgments based on their cognition.

#### 5.1.2. Roles of Familiarity and Concreteness in Symbol Design

Familiarity was the starting point for the application of the framework of symbol design methods based on situational cognitive commonness. It was also an important factor in the formation of cognitive commonness. Among the national standard healthcare symbols in China, some symbols include professional medical tools as the main elements. For example, a single element, a stethoscope, was used to represent the symbol of the “internal medicine department”. As patients in healthcare scenarios, however, older people were generally less familiar with professional medical tools and had a hard time identifying these pictures with a high degree of completeness in their minds. This increased the difficulties in generating the cognitive commonness, resulting in a poor understanding of the existing symbols. In the design practice for healthcare symbols, this study recruited older people with a high familiarity with the healthcare environment as participants to draw the common graphics. Their drawing ideas focused on familiar physical and social situations. For example, the common ideas about the “internal medicine department” obtained by older Chinese people included “stethoscope” and “scene of a patient visiting a doctor” (Figure 4). From the perspective of situated cognition, the designers put “a doctor using stethoscope to examine a patient” into the symbol for the “internal medicine department”. The users were highly familiar with this scenario. Thus, the designed symbol effectively overcame the problem of poor understanding of medical tools (which were used as design elements in the national standard healthcare symbols in China) among older Chinese people. In addition, a poor understanding of the symbols was improved after partial optimization of the symbols, suggesting the role of familiarity was important in symbol design. For example, our optimized symbol for “ultrasound department” consisted of fewer components (which included only an ultrasound screen, a patient, and a bed; see Figure 5 for details) than the national standard healthcare symbols in China (which included an ultrasound screen, a patient, a bed, and a radiologist; see Figure 2 for details). The lack of key elements in the scene may result in the poor understanding of the users. A study by Shen [39] showed that icons that were highly comprehensible tended to represent events and items that the users were familiar with. In the present study, the older patients had a single perception perspective in the limited healthcare scenario. As cognitive commonness generated by high familiarity was amplified, this led to the neglect of other situational elements and resulted in the usage of fewer symbolic elements and a poorer understanding among older users. This finding also suggests that it is necessary to recruit different user populations for a situation when designing a symbol based on situational cognitive commonness in order to enrich the cognitive commonness of specific situations.

Moreover, concrete expression played a key role in acquiring the cognitive commonness of older people and also the design output. The drawings of older people when generating cognitive conception graphics mostly contained concrete elements. For example, the symbols prepared by our participants for “scene of a patient visiting a doctor” in the “internal medicine department” included the drawings of “a doctor”, “patient”, and “table”. This phenomenon of concrete representation was also shown in other graphic drawings, which verified the findings of Ng [17] that people were more likely to create concrete graphics of cognitive commonness. Designers of our study also adopted concrete expression when processing and designing the common graphics. The final test results verified that the understanding of the symbols among older people was improved, which was consistent with the findings of the report by Jones [42] that the concrete design method directly reflected the cognitive commonness of the users.

### 5.2. Application Suggestions

First, we suggest incorporating situated cognition into symbol design processes. Based on the cognitive commonness of specific users to symbols, the optimized symbols may effectively improve the users’ understanding of the symbols. Compared with the previous symbol design methods based on the principle of user participation, the composition of the situation is more conducive to users’ memory of the real scene that the graphics and wayfinding signs are meant to represent. Familiarity can make users provide comprehensive information when generating graphics, and the use of concreteness in the design of the graphics can intuitively express the symbol semantics that the designers wish users to acquire. This method can better obtain the users’ more real and common ideas. It can also help designers implement their ideas more effectively, and therefore make it easier for users to understand symbols. The cognition of each healthcare department among older people generally stems from the physical and social situations with which they interact. Thus, it is necessary to make the composition of symbols align with their understanding by restoring the characters, physical equipment, healthcare environment, and interaction between the patients and the doctors. When designing symbols for complex scenarios, the drawings from users may not completely present all scenarios, as such designers should supplement the situational features in order to prepare symbols that best fit with the actual scenarios.

Second, we suggest selecting participants who are familiar with the target information when applying the framework of symbol design methods that are based on situational cognitive commonness. This should improve the efficiency and quality of common graphic creation and lay a solid foundation for designers to extract common graphics, thereby effectively improving understanding of designed symbols.

Third, designers should try their best to guide users to draw concrete graphics and to process and enhance the concreteness of the design in order to minimize the difficulties of understanding among users. Designers may have operational differences in the extraction and prototyping stages, which may lead to different design results. Designers are required to be familiar with wayfinding target situations in advance; further they must try to understand users’ real drawing ideas in depth and to carry out design practice as well.

### 5.3. Limitations and Future Research

This study does also have some limitations: (1) This study focused on the current situation that older Chinese people have a poor understanding of healthcare wayfinding signs in China. The design practice using the framework of this study was based on healthcare wayfinding signs and the older population. Therefore, the application is most suitable for the healthcare symbol design field targeting the older population. More in-depth research in the future should be conducted on symbols from different fields or that integrate multiple fields. (2) This research paid little attention to the effects of user factors on the generation of cognitive commonness. Older people from different age groups and having different characteristics may have different cognitive patterns. Future studies should include participants from different backgrounds and education level and age groups and with different characteristics in order to improve the generality of the symbols. (3) This study incorporated familiarity and concreteness into the design framework and design practice. We did not, however, evaluate users’ familiarity with the target information or objects that are included in the National Standard of the People’s Republic of China [45] regulating of medical wayfinding symbols, which may have influenced the judgment of participants who had knowledge of these symbols. The degrees of concreteness in the user-drawn graphics or the designers’ designs were also not objectively evaluated. Therefore, a future study should introduce questionnaires to verify the role of familiarity and concreteness through the users’ scoring. (4) This study only compared the changes in the comprehension of medical wayfinding symbols before and after the redesign, without integrating the medical department symbols and other public symbols within the hospital. Therefore, future studies should test the comprehension of medical symbols and general-purpose symbols within the hospital to verify whether users can accurately recognize the medical symbol design scheme among many symbols.

## 6. Conclusions

This study incorporated the theory of situated cognition and cognitive commonness into symbol design. We proposed a design framework for signage in geriatric hospitals based on older users’ situational cognitive commonness in order to improve the understanding of healthcare and hospital signage among older Chinese people. Using the design practice of nine national standard healthcare wayfinding signs, the design approach in this study further developed the previous symbolic design approach that was based on the principle of user participation. Further, this approach then incorporated new design methods in the design framework in order to address its shortcomings in the interpretation of accurately extracting cognitive commonness with: the composition of the situation that enhanced the real common ideas, the familiarity that promoted users to recall situations comprehensively, and the concreteness that expressed the semantics of symbols intuitively, which all improved the understanding of the medical wayfinding symbols among older people. This study provided a feasible theoretical basis that can be used to improve the graphic design of wayfinding signs for older people in a healthcare environment. It can also serve as a research reference in the graphic design for public environments in order to improve the understanding of signs.

## Figures and Tables

**Table 1:** Paired *t*-test results of symbols.

Symbol	Degree of Understanding (Mean ± Standard Deviation)		Difference	*t*
	Design	Existing		
Internal medicine department	73.14 ± 20.95	53.26 ± 24.96	19.88	6.033 ***
Surgery department	65.82 ± 21.15	50.38 ± 21.68	15.44	6.010 ***
Radiology department	66.62 ± 24.55	50.60 ± 25.19	16.03	4.093 ***
Nurse	64.09 ± 27.02	50.69 ± 24.79	13.4	3.212 **
Observation room	70.25 ± 23.60	55.55 ± 25.07	14.7	3.976 ***
Gynecology department	61.64 ± 24.50	55.30 ± 22.16	6.34	2.039 *
Western pharmacy	64.47 ± 21.82	63.30 ± 25.29	1.17	0.363
TCM pharmacy	63.82 ± 22.30	63.82 ± 23.48	0	0
Ultrasound room	59.87 ± 24.13	65.09 ± 26.62	−5.22	−1.295

* *p* < 0.05, ** *p* < 0.01, and *** *p* < 0.001 significance differences.

## Data Availability

Not applicable.
